# Trade‐offs in moving citizen‐based anuran call surveys online during the SARS‐CoV‐2 pandemic: Lessons from rural Appalachia, USA

**DOI:** 10.1002/ece3.6654

**Published:** 2020-08-15

**Authors:** Walter H. Smith, Michael Kevin Hamed

**Affiliations:** ^1^ Department of Natural Sciences The University of Virginia's College at Wise Wise Virginia USA; ^2^ Department of Fish and Wildlife Conservation College of Natural Resources and the Environment Virginia Polytechnic Institute and State University Blacksburg Virginia USA

**Keywords:** citizen science, ecology, Mountain Chorus Frog, Virginia, wetland

## Abstract

Citizen science approaches provide adaptable methodologies for enhancing the natural history knowledge of understudied taxa and engaging underserved populations with biodiversity. However, transitions to remote, virtual training, and participant recruitment in response to public health crises like the SARS‐CoV‐2 pandemic have the potential to disrupt citizen science projects. We present a comparison of outputs from a citizen science initiative built around call surveys for the Mountain Chorus Frog (*Pseudacris brachyphona*), an understudied anuran, in Appalachian Virginia, USA, prior to and during the SARS‐CoV‐2 pandemic. A transition to virtual training in this initiative did not lead to a decrease in scientific output and led to unexpected natural history insight about our focal taxon; however, a reliance on virtual instruction did decrease overall participation by local residents, particularly for rural K‐12 students. We discuss the trade‐offs exhibited by the adaptation of our initiative to a virtual format and provide recommendations for other citizen science initiatives facing similar restrictions in the face of current and future public health crises.

## Graphical abstract

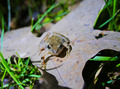

## 1 INTRODUCTION

Citizen science approaches are becoming increasingly integrated into biological inventory protocols and basic survey efforts for various taxa. This is particularly true for easy‐to‐identify taxa that are located across undersurveyed areas and/or regions dominated by privately owned lands that are inaccessible to biologists (Dickinson, Zuckerberg, & Bonter, 2010; Kosmala, Wiggins, Swanson, & Simmons, 2016). Citizen science projects now enjoy widespread use across multiple ecological and evolutionary subdisciplines, involving members of the public in myriad research and outreach efforts (Hochachka et al., 2012; Pocock, Tweddle, Savage, Robinson, & Roy, 2017; Worthington et al., 2011).

Beyond benefits related to enhancing data collection for researchers, citizen science projects are also increasingly being used to supplement or enhance instructional strategies for science educators (Shah & Martinez, 2016). The inquiry‐based learning methods employed by citizen science projects can expose students to facets of project design, data collection, and data curation in a real‐world setting (Kountoupes & Oberhauser, 2008; Oberhauser & LeBuhn, 2012), while engagement with citizen science projects has been shown to both give participants a voice in public decision‐making processes and increase participants’ understanding of high‐level scientific concepts (Ballard, Dixon, & Harris, 2017; Bonney, Phillips, Ballard, & Enck, 2015; Green & Medina‐Jerez, 2012). These educational benefits to participants hold especially true when researchers are intentional about incorporating science learning into citizen science projects and build those projects around clearly defined educational needs (Pandya & Dibner, 2018).

Anurans are especially well suited to citizen science monitoring due to their characteristic advertisement calls that can be used by observers to identify the presence and relative abundance of common species, often with minimal training (Crouch & Paton, 2002; Shirose et al., 1997). Citizen science‐based anuran call surveys have enjoyed widespread use by academic researchers and government agencies for decades (Bishop et al., 1995; Mossman, Hartman, Hay, Sauer, & Dhuey, 1998; Smit, Zuiderwijk, & Groenveld, 1999), leading to advancement in our knowledge of anuran diversity and anuran species’ ecological and evolutionary dynamics. Such datasets, for example, have clarified the local and regional distributions of individual species (Cunningham, Davis, Swarth, & Therres, 2012; Rowley et al., 2019) and informed the broader understanding of conservation concerns for anuran diversity (Cosentino et al., 2014; Westgate et al., 2015). Anuran call surveys and their resulting datasets have also been widely used as a medium for incorporating citizen science into curricula across multiple educational levels, including public (K‐12) schools and undergraduate classrooms (Cosentino et al., 2014; Huffling et al., 2018; Kim, Sung, Park, & Park, 2006).

Large‐scale public health crises like the SARS‐CoV‐2 outbreak have the potential to disrupt citizen science initiatives, including anuran call surveys, that rely upon the training of citizen observers prior to the onset of data collection. Social distancing requirements and associated health concerns may limit or entirely preclude in‐person training sessions for community‐based initiatives, although remote instruction may provide one avenue for addressing public health limitations while allowing some portions of community‐based programs to remain intact (Iyengar & Shin, 2020). However, little information currently exists examining the trade‐offs of such a transition in instruction for both citizen observers and data resulting from such initiatives.

Beginning in 2019, we launched a citizen science initiative aimed at involving residents of southwest Virginia, USA—a rural portion of the Appalachian Mountains that has been historically underserved by scientific outreach and education efforts (Haight & Gonzalez‐Espada, 2009)—in anuran call surveys for the Mountain Chorus Frog (*Pseudacris brachyphona),* an easily identified yet understudied anuran with high regional conservation priority (VDGIF, 2015). The SARS‐CoV‐2 outbreak of early 2020 occurred just prior to the start of this initiative's second year, providing a unique opportunity to examine the output of our initiative prior to and after disruption by the outbreak and a transition to virtual training for survey participants. Here, we compare the results of our initiative before and after this transition, and outline trade‐offs for both researchers and participants and discuss the relevance of these findings to other citizen science approaches.

## 2 MATERIALS AND METHODS

### 2.1 Focal taxon and original project design

**FIGURE 1:**
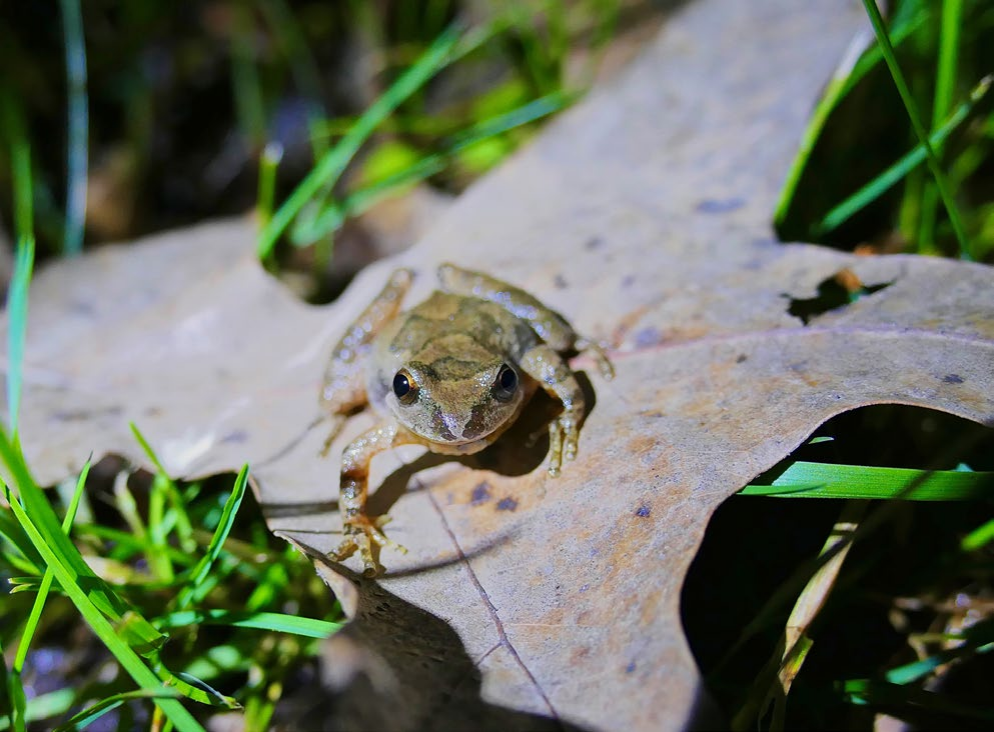
A male *Pseudacris brachyphona* during advertisement calling activity in a small, isolated wetland in southwest Virginia, USA

The Mountain Chorus Frog (*Pseudacris brachyphona*) is a small member of Hylidae adapted to forests across portions of the eastern United States, primarily centered across the southern and central Appalachian Mountains (Figure 1). Mountain Chorus Frogs breed in late winter and early spring in small, shallow, and isolated wetlands, including both naturally occurring and artificial wetland features (Green, 1938). Despite having a distinct and easily recognizable advertisement call, large portions of the species’ range suffer from data deficiency that precludes the design of larger ecological and evolutionary studies, with rankings reflecting high conservation priority in several states (NCWRC, 2014; PFBC, 2015; VDGIF, 2015).

Beginning in January 2019, we launched a citizen science initiative aimed at inventorying Mountain Chorus Frogs across the Upper Tennessee and Ohio River drainages in southwest Virginia, USA—an area lacking a history of intensive inventory and monitoring for this species. Our initiative had two goals: (a) increase the distributional knowledge of Mountain Chorus Frogs across the study area and (b) engage rural residents from historically underserved communities, particularly K‐12 students and undergraduates, with anuran biodiversity and associated monitoring approaches. We initially trained 486 individuals (446 students/educators and 40 members of amateur naturalist groups) through in‐person programs from February–May 2019, corresponding to the period just prior to and during the species’ local breeding season. The locations of in‐person training modules were chosen to overlap with the known distribution of Mountain Chorus Frogs in Virginia.

Training modules consisted of presentations by the coauthors on regional anuran diversity, characteristics of Mountain Chorus Frogs, the purposes of the monitoring effort, and how to identify the species’ advertisement call and distinguish it from other regional taxa. When possible, we accompanied participants to local wetland habitats on public lands following training events to model field protocols. All participants were trained to record samples of advertisement calls as audio or video files using their smartphone's native applications or a digital recorder provided to each participating classroom, along with data on the context of the observation (time, date, geographic coordinates, and weather conditions). Participants then sent observations to the investigators via email. Training information, along with links to online audio samples, was also housed on a website created to allow participants to review training content following the delivery of in‐person modules (https://www.mtchorusfrog.fishwild.vt.edu/). Samples of content used during training modules (organismal information, identification instructions, audio samples, and instructions for recording field observations) can be viewed under the “Learn” and “Report Observations” tabs at the aforementioned website. All approved institutional protocols involving the use of human subjects were followed during the design and implementation of training and data collection procedures.

### 2.2 SARS‐CoV‐2 modifications and comparisons of results

Mandated social distancing requirements, widespread school closures, and associated public health concerns made it impossible for us to continue in‐person training modules for our initiative's second year in spring 2020. Instead, we adapted our training to consist of an asynchronous module recorded via Zoom; this module and the aforementioned website housing training content was then shared with educators and the public via email and social media (Facebook, Twitter) to recruit interested participants. Participants were encouraged to only participate in sampling if it was possible to meet social distancing requirements (avoiding closed public lands, avoiding large groups, and maintaining a minimum of six feet between observers in the field). For both years of the project, we accepted submissions from citizen observers throughout the entirety of the species’ local breeding season (roughly 1 March to 1 June), with participants encouraged to sample accessible public lands and only those private lands that participants either owned or had permission to sample. 434 individuals viewed our virtual training video during the 2020 field season, providing a similar scope of trainees across both years of the initiative.

We hypothesized that a switch to virtual, asynchronous training and social distancing restrictions imposed on participants as a result of the SARS‐CoV‐2 outbreak would: (a) result in fewer overall citizen submissions, (b) reduce the proportion of reports submitted from public lands, relative to those recorded on participants’ private property, and (c) increase the rate of misidentified advertisement calls due to limited training and a lack of field‐based engagement with the project investigators. We assessed these differences following the completion of the species’ peak local breeding season in June 2020.

## 3 RESULTS

**FIGURE 2:**
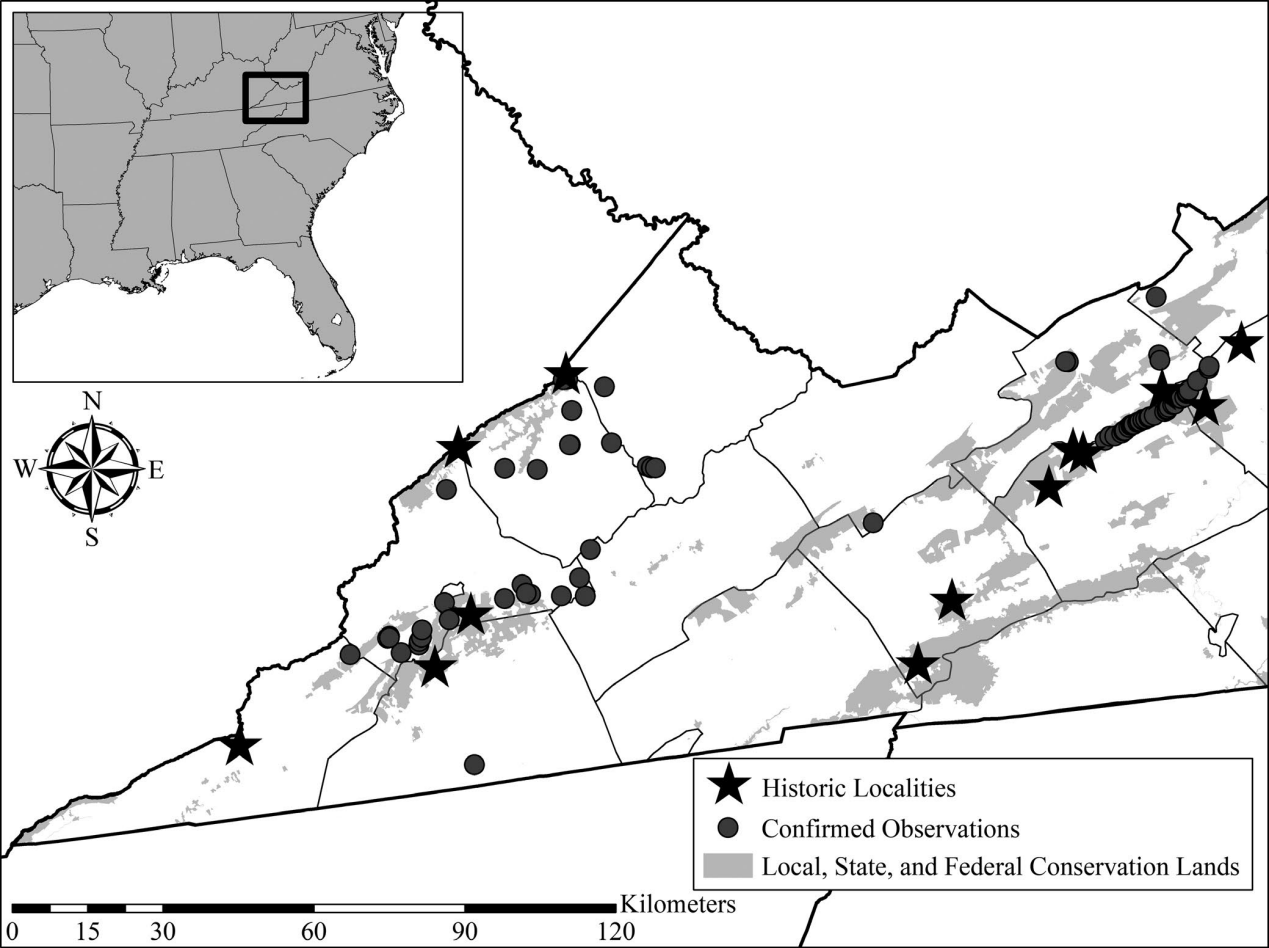
Location of the study area across multiple counties in southwest Virginia, USA, showing historic records of *Pseudacris brachyphona* prior to 2019 and confirmed citizen science observations from 2019 and 2020. Black box in inset map denotes the location of the study area

Citizen observers across both years reported a total of 83 confirmed observations of Mountain Chorus Frogs backed by verifiable sound recordings or photographs, which accounted for nearly six times the number of historic observations recorded by biologists from the late 1800s to 2019 across the study area (*N* = 14; Figure 2). These observations included range expansions for the species across one Virginia county. Thirty observations were reported in 2019, with 53 observations reported during the SARS‐CoV‐2 outbreak in 2020. The number of observations per participant increased from 2019 to 2020, while the number of student participants dropped substantially from 2019 to 2020, with increased participation from members of amateur naturalist groups (Table 1).

**TABLE 1:** Total confirmed observations of *Pseudacris brachyphona*, observations per participant, participant backgrounds, and confirmed observations by habitat type for 2019 (prior to adjustment due to SARS‐CoV‐2) and 2020 (following adjustment due to SARS‐CoV‐2)

	2019	2020
Observations and participants		
Total observations	30	53
Observations per participant	1.9	6.6
Student participants (%)	46.7	7.5
Amateur Naturalist participants (%)	53.3	92.5
Observations by habitat type		
Former surface mine	3.3	0
Gas well infrastructure	6.7	0
Natural wetland, private	13.3	20.8
Natural wetland, public	26.7	5.6
Roadside ditch, private	30.0	71.7
Roadside ditch, public	20.0	1.9

Note

Data for habitat types are percentages of total observations for that year; “public” and “private” refer to observations from public and private lands, respectively.

The locations of citizen‐reported frog localities also shifted from 2019 to 2020. Observations in 2019 were primarily reported from large parcels of public land across the study region, including the Jefferson National Forest, a park jointly managed by the state of Virginia and Kentucky (Breaks Interstate Park), and several state‐managed multi‐use trail systems. By contrast, observations in 2020 were primarily reported from privately owned lands (Table 1). Private lands in both years included natural and artificial wetlands at private residences, flooded ditches along secondary roads, and wetlands formed as a result of infrastructure (access roads, well pads, and mine benches) developed for coal and gas extraction or agricultural activities.

False positives, or reports of species other than Mountain Chorus Frogs, were minimal both prior to and during the SARS‐CoV‐2 outbreak (6.7% and 1.9% of observations, respectively). Misidentified observations were primarily constrained to congeners that are common and abundant in the study region, including Spring Peepers (*Pseudacris crucifer*; *n* = 1 observation) and Upland Chorus Frogs (*Pseudacris feriarum*, *n* = 1 observation), as well as Cope's Gray Treefrog (*Hyla chrysoscelis*, *n* = 1 observation).

## 4 DISCUSSION

Contrary to our predictions, a switch to virtual training and the presence of social distancing restrictions imposed on participants did not result in a decrease in reported observations of Mountain Chorus Frogs between years prior to and during the SARS‐CoV‐2 outbreak, despite fewer individuals being reached by training materials during the outbreak. Instead, reported observations increased during the outbreak, without a corresponding increase in misidentified anuran observations in light of reduced in‐person training. These results are promising since they suggest that relying on remote and asynchronous recruitment and training alone do not necessarily weaken the output of some citizen science initiatives.

We also observed a shift in the location of reported observations from project participants that provided new and unexpected insight into the natural history of our focal species. Citizen reports shifted from being primarily focused on public lands in 2019 to private lands in 2020 during the SARS‐CoV‐2 outbreak. While the remote nature of our initiative in 2020 prevented us from robustly assessing participants’ motivations for selecting sampling sites, it is plausible that the shift we observed occurred in response to land access restrictions and public health recommendations coinciding with the sampling period. Our study region, for example, experienced widespread closures of public land access points, as well as social distancing recommendations from public officials that encouraged residents to stay at home and avoid public settings during the outbreak, which peaked regionally alongside the peak local breeding season for Mountain Chorus Frogs (Virginia Department of Health, 2020).

The aforementioned shift to sampling private lands provided unexpected benefits to understanding the natural history of Mountain Chorus Frogs. Specifically, participants recorded several observations of Mountain Chorus Frogs using small, isolated wetlands formed incidentally as a result of the development of infrastructure for coal and gas extraction operations during the project's first year. Wetlands formed alongside access roads, on mine benches, and at gas well pads all provided numerous Mountain Chorus Frog observations. These habitats are common in private inholdings along public trail systems and public land access roads across our study region, and many individuals reporting observations from these habitats encountered calling frogs while visiting adjacent public lands.

The use of artificially created wetlands by Mountain Chorus Frogs has been previously reported (Drayer & Richter, 2016; Green, 1938), although the species’ apparent abundance in wetlands created as a result of extractive industry infrastructure appears to be novel and has implications for the population dynamics and distribution of this species across the Appalachian coalfields, where landscape change as a result of resource extraction is occurring at an increasing rate (Drohan, Brittingham, Bishop, & Yoder, 2012; Townsend et al., 2009). Future studies targeting these habitats may be able to clarify the species’ affinity for such sites, as well as identify specific habitat characteristics preferred by frogs in such incidentally created wetlands.

By contrast, observations submitted in 2020 following our adjustment to virtual training were almost exclusively reported from roadside ditches and wetlands adjacent to areas characterized by agricultural land use. This shift was likely due to participants refraining from visiting public lands and searching for frogs primarily in and around their own properties and places of residence. Wetlands associated with agricultural land use are well‐known habitats for members of *Pseudacris* (Barbour, 1957; Knutson et al., 2004); however, little attention has been given to these habitats in Appalachian Virginia and portions of adjacent states. The apparent abundance of Mountain Chorus Frogs in agricultural areas reported by participants in our study presents important information for understanding the species’ regional natural history, as well as for the future design of management and conservation strategies targeted at engaging private landowners. More broadly, these results show that the adaptation of citizen science projects in light of public health concerns can lead to new scientific insight that can generate novel hypotheses for future study, without decreasing the project's overall output.

We did, however, also record several key trade‐offs following the SARS‐CoV‐2 outbreak and related modifications to our initiative. While the number of overall observations reported through our initiative increased from 2019 to 2020, this increase was due to fewer overall participants reporting more observations per participant than prior to the outbreak. In addition, the proportion of educators and students reporting observations dropped dramatically, shifting almost exclusively to members of amateur naturalist groups during the SARS‐CoV‐2 outbreak. Despite the increased scientific knowledge gained during this time period, these results point to disadvantages of our modified program structure, specifically related to our goals of increasing engagement among rural and underserved student populations.

One limitation of our adapted program during the pandemic is that it lacked a robust assessment structure designed to gauge the motivations of project participants and their limitations in participating in the virtual version of our initiative. This constrains our ability to definitively assess why student participation dropped following a switch to online training, although this was likely due to the existing socioeconomic context of our study region. Central Appalachia is home to significantly lower levels of Internet access than the general United States population, with recent estimates suggesting that less than 60% of households have access to the Internet in most counties across our study area (Pollard & Jacobsen, 2019). Despite enthusiastic responses from educators following our switch to online training, it is likely that many of those educators’ students simply could not be reached by our virtual training module due to a low rate of regional Internet access and an associated reliance on hardcopy instructional materials by educators for remote instruction during the SARS‐CoV‐2 outbreak.

The decrease in student engagement and overall participation observed during the pandemic—in spite of an increase in the amount of total submitted observations—highlights a key trade‐off in the use of citizen science initiatives. Effective citizen science programs should not simply be tools for enhancing data collection but should also maximize engagement with target populations and interaction with professional scientists (Bonney et al., 2015; Dickinson et al., 2010). Technological constraints within the target population may present equity issues for such engagement, especially when initiatives are designed for low‐income, remote, or underserved populations (Carballo‐Cardenas & Tobi, 2016; Hobbs & White, 2012). Researchers should keep these considerations and possible solutions in mind when adapting existing initiatives to a virtual format in the face of public health issues like the SARS‐CoV‐2 pandemic. For our initiative, one such solution may be to provide hardcopy training materials to educators who are engaged with students without Internet accessibility, as well as interviewing educators to better ascertain their instructional needs and the limitations of their respective student populations prior to designing remote training modules.

More broadly, our results highlight the need for researchers to consider potential trade‐offs associated with adapting citizen science approaches in response to large‐scale public health concerns. While virtual training and engagement can still provide scientific benefits under such scenarios, substantial considerations exist with respect to the equity and inclusiveness of public initiatives relying on remote, virtual instruction, as well as how those initiatives can best develop assessment approaches during transitions in their engagement strategies to identify and address such concerns. These considerations will be key for citizen science programs during future waves of the SARS‐CoV‐2 pandemic and other public health scenarios requiring similar adjustments to program structure.

## CONFLICT OF INTEREST

None declared.

## AUTHOR CONTRIBUTION

**Walter Smith:** Data curation (equal); Formal analysis (lead); Funding acquisition (supporting); Methodology (equal); Writing‐original draft (lead); Writing‐review & editing (equal). **M. Kevin Hamed:** Data curation (equal); Formal analysis (supporting); Funding acquisition (lead); Methodology (equal); Writing‐original draft (supporting); Writing‐review & editing (equal).

## Data Availability

The full dataset of citizen observations recorded through this project was uploaded to the Dryad data repository (https://doi.org/10.5061/dryad.6wwpzgmwc). Data were also provided to the Virginia Department of Wildlife Resources for archiving in its public Virginia Fish and Wildlife Information Service repository (https://vafwis.dgif.virginia.gov/fwis/; Species Code 020011).
